# Which Combined Profiles of Physical Activity and Dietary Intake Are Associated with Postpartum Prediabetes Status Among Women with Prior Gestational Diabetes Mellitus in Underserved Rural Areas of Central South China?

**DOI:** 10.3390/nu18050812

**Published:** 2026-03-01

**Authors:** Mengdi Li, Qingqing Liu, Yao Chen, Yimeng Li, Zhenzhen Rao, Manping Wang, Carles Muntaner, Jia Guo

**Affiliations:** 1 Xiangya School of Nursing, Central South University, 172 Tongzipo Road, Changsha 410013, China; 237811004@csu.edu.cn (M.L.); 247801021@csu.edu.cn (Q.L.); 227801001@csu.edu.cn (Y.C.); yimenglimuremure@126.com (Y.L.); 2 Department of Epidemiology and Health Statistics, Xiangya School of Public Health, Central South University, 172 Tongzipo Road, Changsha 410013, China; zhenzhenrao@csu.edu.cn; 3 School of Nursing, University of Hong Kong, Hong Kong 100872, China; mpwang@hku.hk; 4 Department of Psychiatry, University of Toronto, Toronto, ON M5T 3M7, Canada; carles.muntaner@utoronto.ca

**Keywords:** dietary intake, physical activity, prediabetic state, prior gestational diabetes mellitus, underserved areas

## Abstract

Background/Objectives: Women with prior gestational diabetes mellitus (GDM) are at higher risk for prediabetes, particularly when inactivity or poor diet persists after childbirth. These behaviors often co-occur, and their combined effect is greater than the sum of individual risks. This study aimed to identify physical activity and dietary profiles among women with prior GDM in underserved areas, examine their association with impaired fasting glucose (IFG) and impaired glucose tolerance (IGT), and investigate their associated factors. Methods: A cross-sectional analysis of baseline data collected in July 2018 and November 2022 from two randomized controlled trials was conducted (*n* = 633). Activity, dietary intake, glucose levels, and socio-demographic, anthropometric, and psychosocial characteristics were collected. Latent profile analysis identified behavior profiles. Binary and multiple logistic regressions assessed associations and influencing factors. Results: Three distinct profiles were identified including “Less Activity and Low Dietary Fiber Intake group”, “Adequate Activity but Low Dietary Fiber Intake group”, and “Adequate Activity but High Starch Intake group”. Compared with the “Adequate Activity but Low Dietary Fiber Intake group”, the “Less Activity and Low Dietary Fiber Intake group” had increased IFG risk (odds ratio [OR], 3.792; 95% CI, 1.146–12.543); women with non-precarious employment, no family history of diabetes, or inadequate external environmental resources were more likely in this group. “Adequate Activity but High Starch Intake group” had higher IFG (OR, 6.321; 95% CI, 1.500–26.639) and IGT (OR, 6.030; 95% CI, 1.530–23.770) risk; women with family income <416 USD/month or worse psychological health tended toward this group. Conclusions: Unhealthy behavior profiles were observed among women with prior GDM. High starch intake and insufficient activity were associated with greater prediabetes risks. Screening and education on physical activity and diet may warrant particular attention among women with non-precarious employment, low family income, or no family history of diabetes. In addition, integrating strategies that enhance psychological health and improve external environmental resources into lifestyle-related interventions may represent a promising approach.

## 1. Introduction

Gestational diabetes mellitus (GDM) is defined as hyperglycemia first recognized during pregnancy [1]. The prevalence of GDM varies globally, with higher rates reported in underserved regions such as Africa and Southeast Asia (14.2–20.8%) compared to developed areas like Europe and North America (7.1–10.4%) [2]. Women with prior GDM face a nine- to ten-fold higher lifetime risk of developing type 2 diabetes mellitus (T2DM) compared to those without GDM [3,4]. However, on the one hand, many women often overlook this risk after childbirth [5]; on the other hand, there is a lack of clear direction as to who should bear the responsibility of postpartum care for women [6]. Prediabetes status, including impaired fasting glucose (IFG) and impaired glucose tolerance (IGT), represents a significant transitional phase that precedes T2DM [7]. Women with prior GDM exhibit as much as a 50.9% likelihood of developing prediabetes status within two years post-childbirth, which is more than double the rate observed in women without prior GDM [8,9]. Therefore, the early identification of modifiable risk factors related to prediabetes status is crucial, particularly in underserved regions.

The World Health Organization has identified the promotion of healthy lifestyle behaviors as one of the most cost-effective strategies for preventing T2DM [10]. Among these behaviors, physical activity and dietary intake are pivotal modifiable factors [11]. Extensive evidence has demonstrated that either insufficient moderate-to-vigorous physical activity or a diet deficient in fruits and vegetables is associated with an elevated risk of postpartum prediabetes status among women with prior GDM [12,13,14]. In underserved areas, these detrimental behaviors also play a significant role in expediting this progression among this population [15,16]. Furthermore, numerous researchers have posited that detrimental behaviors are interconnected and frequently manifest concurrently [17]. Previous systematic reviews have indicated that the combined impact of detrimental behaviors elevates the risk of diabetes beyond the mere aggregation of individual risks [18,19]. Nevertheless, there exists a paucity of research that has explored the combined effects of these behaviors specifically in women with prior GDM. An exhaustive understanding of combined physical activity and dietary intake, in conjunction with its correlation to prediabetes status, is critically required.

Recognizing modifiable risk factors associated with the combined physical activity and dietary intake profiles is crucial for developing targeted interventions to reduce the risk of prediabetes status. Previous studies have suggested that women with prior GDM often encounter various barriers to developing or maintaining a healthy physical activity and dietary intake, including financial constraints, negative emotions, lack of time, and limited resources [20,21,22]. There remains a substantial gap in the literature regarding the specific factors that influence physical activity and diet intake among this population in underserved areas. The socio-ecological model (SEM) provides a comprehensive framework for understanding how behaviors are determined by factors at multiple levels, including the individual, interpersonal, community, and societal levels [23]. It can also help systematically examine factors associated with these high-risk physical activity and dietary intake profiles.

Taken together, we aimed to (1) identify physical activity and dietary intake profiles among women with prior GDM in underserved rural areas of Central South China [24], (2) explore the association between these profiles and IFG as well as IGT, and (3) investigate the factors related to these profiles that are associated with IFG or IGT with the guidance of SEM.

## 2. Materials and Methods

### 2.1. Study Design

This study was a cross-sectional study. All data were derived from baseline data of two randomized controlled trials conducted between 2018 and 2019, and between 2022 and 2023, respectively. All baseline data were collected prior to randomization and intervention implementation in July 2018 and November 2022, respectively. No intervention effects were evaluated in the present analysis. These two randomized controlled trials aimed to reduce the prevalence of diabetes among women with prior GDM and were registered in the Chinese Clinical Trial Registry (ChiCTR1800015023 and ChiCTR2200058150) [24,25]. Ethics approval has been obtained from the Ethics Review Committee (No. 2016034 and E2021162). This study was reported in accordance with the Strengthening the Reporting of Observational Studies in Epidemiology (STROBE) Statement.

### 2.2. Setting, Participants, and Sampling

The research was carried out in two rural regions of Hunan Province, located in Central South China: You County and Yongding County, each representing distinct geographic locations, socioeconomic statuses, and ethnic groups. You County is situated in the southeastern part of Hunan and has a predominant Han Chinese population, constituting 90% of its demographic, along with a GDP that exceeds the provincial average. Conversely, Yongding County is inhabited by ethnic minorities in the northwestern region of Hunan and exhibits a moderate GDP per capita. Rural areas within China commonly experience a deficiency in primary health care resources and primary care providers, which aligns with the fundamental definition of underserved areas [26]. Consequently, for the purposes of this study, these two rural areas in Hunan Province are designated as underserved areas.

All participants were recruited at the General Hospital of You County and the Maternal and Children’s Hospital of Yongding County. These two hospitals are situated in the centers of their respective counties, facilitating the provision of delivery and postnatal health care services to most women in You County and Yongding County. Eligible participants were required to meet the following criteria: (a) a documented history of GDM; (b) being over 18 years of age; (c) being at least 6 weeks postpartum and no more than 3 years postpartum; (d) a commitment to residing in the research counties for a minimum of 3 years; and (e) possessing access to a smartphone. The exclusion criteria were (a) intending to conceive within the upcoming three years; (b) currently pregnant; (c) having been diagnosed with diabetes before pregnancy or after delivery; (d) undergoing treatment with medications that affect glucose metabolism; and (e) having experienced multiple pregnancies (defined as three or more).

A post-hoc power analysis was conducted using PASS 2021 to determine the required sample size for the binary logistic regression analysis. The analysis showed that a sample size of 633 provided sufficient power, with minimum β values of 0.9843, indicating that this sample size is adequate for detecting true effects.

### 2.3. Data Collection

The researchers obtained and utilized baseline data from the original databases of the two randomized controlled trials conducted in the previous period, which mainly included physical activity, dietary intake, glycemic status, demographic characteristics, anthropometric characteristics, and psychosocial characteristics.

### 2.4. Variables and Measures

We examined the association between physical activity and dietary intake profiles (as independent variables) and prediabetes status (with IFG or IGT as dependent variables) in women from these underserved areas. We then examined the factors related to these profiles (as dependent variables) that were associated with IFG or IGT by analyzing socio-demographic, anthropometric, and psychosocial characteristics (as independent variables), based on the SEM structure in this population.

Physical activity, including moderate or vigorous activity as well as sitting, was measured using the International Physical Activity Questionnaire Short Form (IPAQ-SF). The IPAQ-SF consists of seven questions designed to capture the average daily time spent sitting, walking, and participating in both moderate and vigorous physical activity over the past seven days. IPAQ-SF has been extensively used, and its reliability is well-documented (Intraclass Correlation Coefficient (ICC)  =  0.79) [27].

The dietary intake, including staple foods, legumes, vegetables, fruits, and dairy products, was assessed using the validated semi-quantitative Food Frequency Questionnaire (FFQ), which was developed based on the dietary guidelines for Chinese residents and the National Health and Dietary Survey in China. Participants were asked how frequently (the number of times per day, per week, per month, per year, or never) and in what quantity (grams or milliliters) they consumed the food over the past three months. A color food photography atlas featuring different portion sizes of all food items was provided to help make the estimation more accurate. Previous studies have shown that this FFQ demonstrates good validity and reliability for Chinese adults, with correlation coefficients between this FFQ and averages from 24-h diet recalls ranging from 0.43 to 0.67 for major food groups [28].

Prediabetes status: IFG and IGT. A standard 2-h 75 g oral glucose tolerance test (OGTT) was performed in all participants. The OGTT was performed in the morning after an overnight fast. Venous blood samples were drawn by a nurse to measure the fasting plasma glucose level (FPG) at fasting and the 2-h plasma glucose level (2 h PG) at 2 h after ingestion of the glucose load. All detection indices of blood samples were analyzed using the hexokinase method with an automated biochemical analyzer in the central laboratory. IFG and IGT were defined according to the 2006 World Health Organization (WHO) criteria. The FPG level from 6.1 mmol/L to 6.9 mmol/L was defined as IFG, and the 2 h PG level from 7.8 mmol/L to 11.0 mmol/L was defined as IGT [29].

Socio-demographic Variables: a self-designed questionnaire gathered data on age, months after delivery, marital status, education, occupation, monthly family income, and family history of diabetes. Based on local socio-demographic and economic characteristics, age was divided into <35 and ≥35 years old based on different body function levels; educational levels were divided into two categories: finishing nine-year compulsory education and not finishing nine-year compulsory education. Occupation was categorized into two main groups: “precarious employment” and “non-precarious employment”, with “precarious employment” defined as job insecurity, inadequate income, and a lack of rights and protections [30]. In our study, farmers, manual laborers, and unemployed individuals were classified as being in the “precarious employment” group due to their lack of stable income and absence of employment-related social security. In contrast, office staff and service sector employees were categorized as part of the “non-precarious employment” group. The monthly family income was classified into two categories: ≥416 USD and <416 USD based on whether women achieved a basic standard of living or not.

Anthropometric Variables: body mass index (BMI) and waist circumference. BMI was calculated as weight in kilograms divided by height in meters squared. Weight (with an empty stomach, light indoor clothing, and no shoes) was measured with weight scales with a precision of 0.1 kg, and height was measured with a stadiometer from the crown of the head to the bottom of the heels, which could be read to 0.1 cm. BMI categories were obesity (≥28 kg/m^2^), overweight (24–27.9 kg/m^2^), and normal (<24 kg/m^2^). Waist circumference was measured with soft, non-stretchable tape with a precision of 0.1 cm, midway between the ribs and iliac crest, and predicted abdominal obesity at 85 cm or more.

Psychosocial Variables: psychological health, social relationships, and external environment were assessed using the World Health Organization Quality of Life-BREF (WHOQOL-BREF) questionnaire [31]. This questionnaire assesses psychological health, social relationships, and environmental conditions through domain-specific scores. The psychological health domain evaluates an individual’s mental well-being and cognitive functioning; the social relationships domain captures aspects of personal intimacy and perceived social support; and the environmental domain primarily reflects the quality and safety of the individual’s external living environment. Higher scores in these three domains indicate better psychological health, social relationships, and environmental status. Additionally, this study found that the Cronbach’s alpha reliability coefficient for this questionnaire was 0.810.

### 2.5. Ethical Consideration

This secondary analysis targeting the association of physical activity and dietary intake profiles with prediabetes status was conducted from 1 December 2024 to 10 January 2025. Given the deidentified nature of the data, specific ethical approval was not required.

### 2.6. Data Analysis

For data organization and description, a total of 17 participants (2.7% of the sample) had missing data in at least one variable. Given the low proportion of missingness (<5%), we addressed missing data using regression methods [32,33], which predicted missing data based on the relationship between the variable with missing data and other variables in the dataset. Continuous variables were presented as mean and standard deviation (SD), and categorical variables were presented as frequency counts and percentages.

LPA was employed to identify the latent profiles of lifestyle behaviors [34], using a finite mixture modeling approach to detect profiles of observations across similar indicators [35]. The mean daily dietary intakes (staple foods, legumes, vegetables, fruits, and dairy products), the average daily time spent on moderate or vigorous physical activity, and the average daily sitting time were included as indicators. We tested models ranging from two to five profiles, and the optimal models were indicated by the following model fit indices [36]: (1) a lower Akaike Information Criterion (AIC), a lower Bayesian Information Criterion (BIC), and lower adjusted-BIC values showing that the model fits well; (2) the higher the entropy value (ranges from zero to one), the higher the classification accuracy; and (3) significant Lo–Mendell–Rubin likelihood ratio test (LMR) and bootstrapped likelihood ratio test (BLRT) values indicated that the k-class model had a better fit than the k-1 model.

The binary logistic regression was used to identify which profiles were associated with IFG and IGT, respectively. Socio-demographic variables, including age, months after delivery, monthly family income, occupation, educational levels, family history of diabetes, BMI, and waist circumference, were considered as covariates in the binary logistic regression [37,38]. Trial was additionally included as a covariate to control for potential between-trial differences. To assess whether the associations differed by trial, interaction terms between lifestyle profiles and trial were further included in the models. Additionally, sensitivity analyses were conducted using complete-case data to evaluate the robustness of the imputed models. The Chi-square test, the Kruskal–Wallis H test, and multiple logistic regression were utilized to investigate factors related to the profiles that were statistically associated with IFG or IGT. The Chi-square test and the Kruskal–Wallis H test were conducted to examine the differences in socio-demographic characteristics, anthropometric characteristics, and psychosocial characteristics among the profiles. Then, significant factors from univariate tests were included in multinomial logistic regression to examine their impact on lifestyle profiles.

The LCA was conducted using Mplus version 8.3. All other analyses were conducted using SPSS 27.0. Two-sided *p*-values less than 0.05 were considered to indicate statistical significance.

## 3. Results

### 3.1. Participant Characteristics

#### 3.1.1. Socio-Demographic, Anthropometric, and Psychosocial Characteristics of the Participants

A total of 633 women with prior GDM participated in this study. Of the 633 participants, 320 (50.55%) were recruited from the randomized controlled trial conducted between 2018 and 2019, and 313 (49.45%) were recruited from the trial conducted between 2022 and 2023. The mean age of the women was 32.78 years (SD = 4.93), and the mean duration since delivery was 17.24 months (SD = 13.58). A total of 627 women (99.05%) were married. A total of 521 women (82.31%) completed nine years of education; moreover, 321 women (50.71%) had precarious employment, while 530 (83.73%) reported a monthly family income of up to 413 USD. Eighty women (12.64%) reported a family history of diabetes. The average BMI was 23.43 (SD = 3.66), and the average waist circumference was 79.47 cm (SD = 9.66). The average scores for psychological health, social relationships, and external environment of the women were 13.56 (SD = 2.02), 14.86 (SD = 2.09), and 13.15 (SD = 1.92), respectively.

#### 3.1.2. Physical Activity and Dietary Intake

Regarding physical activity, the average daily duration of moderate to vigorous physical activity was 56.64 min (SD = 105.94), whereas the average daily sitting time was 247.08 min (SD = 170.61). In terms of diet, the mean daily intakes of staple foods, legumes, vegetables, fruits, and dairy products were 335.79 g (SD = 167.07), 60.32 g (SD = 88.55), 257.31 g (SD = 319.54), 172.45 g (SD = 197.87), and 100.64 mL (SD = 172.59), respectively.

#### 3.1.3. Prediabetes Status

Regarding glycemic status, 67 women (10.58%) had IFG, while 45 women (7.10%) had IGT. The detailed characteristics are displayed in Table 1.

### 3.2. Latent Profile Analysis of the Combined Physical Activity and Dietary Intake

#### 3.2.1. Selecting the Optimal Latent Profile Model

The results of the model fit indices for two to five latent profile models are presented in Table 2. The two-profile model had the highest AIC, BIC, and aBIC values, indicating a poorer model fit and resulting in its exclusion. The four-profile model’s low entropy (<0.8) suggested poor classification accuracy, leading to its exclusion as well. The five-profile model had the lowest AIC, BIC, and aBIC values, indicating the model fit well; however, the entropy value of 0.847 was less than the entropy value of 0.856 in the three-profile model. Thus, the three-profile model has higher classification accuracy than the five-profile model. Based on the theoretical and practical significance, the three-profile model was selected as the optimal behavior profile in our study.

#### 3.2.2. Naming Three Physical Activity and Dietary Intake Profiles

The mean daily dietary intakes (staple foods, legumes, vegetables, fruits, and dairy products), the average daily time spent on moderate or vigorous physical activity, and the average daily sitting time for the three profiles are shown in Table 3.

**Figure 1:**
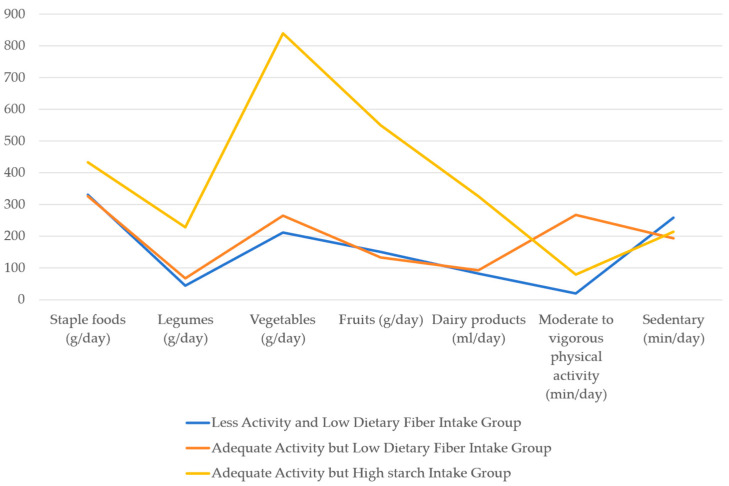
Latent profile plot of physical activity and dietary intake.

Based on the results of our study, we identified three behavior profiles exhibited by women with prior gestational diabetes mellitus in underserved rural areas (Figure 1). The first profile (*n* = 509, 80.41%) is labeled the “Less Activity and Low Dietary Fiber Intake Group” because the average daily time spent on moderate or vigorous physical activity was 20.04 min (SD = 1.37) below the recommendation of 30 min per day [39]. The mean daily intakes of vegetables were 211.75 g (SD = 10.44), and fruits were 150.41 g (SD = 6.86), less than the recommendation of 300–500 g per day and 200–350 g per day [40], respectively. Conversely, the second profile (*n* = 84, 13.27%) is designated as the “Adequate Activity, but Low Dietary Fiber Intake Group” and the third profile (*n* = 40, 6.32%) is referred to as the “Adequate Activity but High Starch Intake Group”.

### 3.3. Association Between the Three Physical Activity and Dietary Intake Profiles and Impaired Fasting Glucose and Impaired Glucose Tolerance

Two binary logistic regression analyses were conducted, adjusting for age, months after delivery, income, education, occupation, diabetes family history, body mass index, waist circumference, and trial, to explore the association of the three profiles with impaired fasting glucose and impaired glucose tolerance, respectively (Table 4). Compared with women classified into the “Adequate Activity but Low Dietary Fiber Intake Group”, women belonging to the “Less Activity and Low Dietary Fiber Intake Group” (odds ratio [OR], 3.792; 95% CI, 1.146–12.543) or the “Adequate Activity but High Starch Intake Group” (OR, 6.321; 95% CI, 1.500–26.639) were significantly more likely to have IFG. Additionally, women belonging to the “Adequate Activity but High Starch Intake Group” (OR, 6.030; 95% CI, 1.530–23.770) were significantly more likely to have IGT. No significant interaction between lifestyle profiles and trial was observed for IFG or IGT (all P for interaction > 0.05, Supplementary Table S1). In addition, sensitivity analyses using complete-case data (*n* = 616) yielded results consistent with the primary imputed analyses (Supplementary Table S2).

### 3.4. The Related Factors of the “Adequate Activity but High Starch Intake Group” and the “Less Activity and Low Dietary Fiber Intake Group”

The three profiles exhibited statistically significant differences in income (χ^2^ = 6.110, *p* < 0.05), family history of diabetes (χ^2^ = 10.333, *p* < 0.05), and occupation (χ^2^ = 7.398, *p* < 0.05). The results of the Kruskal–Wallis H test revealed significant differences in the scores of the psychological health (H = 9.129, *p* < 0.05) and external environment (H = 14.140, *p* < 0.05).

**Figure 2:**
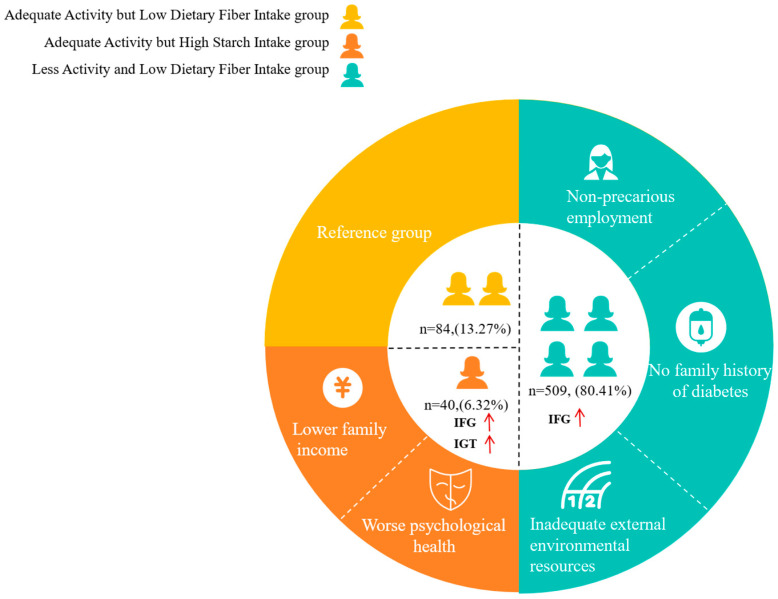
The related factors of the lifestyle group associated with IFG or IGT.

Then, multinomial logistic regression analysis was performed, using the profiles as the dependent variables, with the significant factors identified in the univariate analysis (income, family history of diabetes, occupation, psychological health, and external environment) serving as independent variables. Results showed that (Table 5), in comparison to the “Adequate Activity but Low Dietary Fiber Intake Group”, women with non-precarious employment (OR, 1.898; 95% CI, 1.173–3.102) and those without a family history of diabetes (OR, 1.991; 95% CI, 1.242–4.121) were more likely to belong to the “Less Activity and Low Dietary Fiber Intake Group”. Additionally, women with lower scores in the external environment (OR, 0.776; 95% CI, 0.654–0.920) were also more likely to belong to the Less Activity and Low Dietary Fiber Intake Group. Additionally, women with a monthly family income below 413 USD (OR, 3.817; 95% CI, 1.272–11.450) or poorer psychological well-being (OR, 0.714; 95% CI, 0.556–0.918) were more likely to belong to the “Adequate Activity but High Starch Intake Group” (Figure 2).

## 4. Discussion

In this study, we found that approximately 90% of women in this underserved rural area with combined high-risk behaviors had a co-occurrence of insufficient physical activity and low dietary fiber intake (80.41%), as well as adequate physical activity but high starch intake (6.31%). Inadequate physical activity was associated with an increased risk of IFG. In contrast, adequate physical activity combined with high starch intake was linked to a greater risk of both IFG and IGT. Multiple non-modifiable factors, including occupation, income, and a history of diabetes, were associated with these high-risk profiles. Modifiable factors, such as psychological well-being and external environment, also played a significant role. These findings suggest the potential value of using these non-modifiable characteristics as risk stratifiers, while incorporating the modifiable factors as components of targeted interventions in this population.

The co-existence of insufficient physical activity and low dietary fiber intake emerged as the prime high-risk lifestyle behaviors (80.41%) in this population. This finding aligns with a previous study indicating that various unhealthy behaviors are often interconnected and tend to co-occur [17]. Notably, the rate of co-occurrence of unhealthy physical activity and dietary intake among these women in underserved areas is higher than that of populations in developed regions. For example, in the US, approximately 22% of adults were found to have both poor dietary quality and inadequate physical activity [37]. At the same time, research in Australia also reported that 53% of adults engaged in two or more unhealthy behaviors [41]. Limited resources in underserved rural areas may explain this situation. A shortage of exercise resources, including space, facilities, and instruction, has restricted women’s physical activity [42]. The high cost of healthy foods, particularly fruits and vegetables, is often unaffordable for the majority of the population in underserved areas (i.e., “food deserts”), resulting in a preference for inexpensive, starch-rich processed foods [43].

In our study, the increased risk of IFG observed in women within the “Less Activity and Low Dietary Fiber Intake” group could be explained by the combined negative effects on insulin sensitivity. Reduced physical activity impedes skeletal muscle glucose uptake [44], limiting the ability of muscle cells to clear glucose from the bloodstream, which decreases insulin sensitivity. Low dietary fiber intake also diminishes the production of beneficial short-chain fatty acids (SCFAs) essential for enhancing insulin sensitivity and regulating glucose metabolism [45]. Notably, while this group is associated with a heightened risk of IFG, no significant relationship was observed with IGT. This discrepancy can be explained by the distinct pathophysiological mechanisms underlying IFG and IGT. IFG often reflects an earlier stage of glucose dysregulation, with mild or no beta-cell dysfunction. In contrast, IGT typically indicates a more advanced metabolic disturbance, involving a loss of 50% or more of pancreatic beta-cell function [46,47]. Women in the “Less Activity and Low Dietary Fiber Intake” group were more likely to experience early metabolic changes characterized by reduced insulin sensitivity without significant impairment of beta-cell function. These early metabolic changes are more prone to presenting as IFG rather than IGT, which may elucidate the observed pattern of association.

In contrast, women classified in the “Adequate Activity but High Starch Intake” group showed a significantly higher likelihood of having both IFG and IGT, despite maintaining adequate physical activity and dietary fiber intake. This finding highlights that adequate physical activity alone may not be sufficient to offset the metabolic burden imposed by a high starch intake. Women in underserved areas are more likely to choose refined carbohydrates with a high glycemic index (GI) and rich starch as their primary source of carbohydrates [48,49,50]. High-GI foods rapidly elevate blood glucose levels, causing women with high starch intake to experience sudden spikes in blood glucose, which the pancreas counteracts by releasing insulin [51]. Frequent glucose spikes can damage pancreatic beta cells [52], thereby diminishing insulin production, while the repeated stimulation of insulin receptors by elevated insulin concentrations may increase insulin resistance [53]. These mechanisms disrupt the body’s natural glucose regulation, leading to elevated glucose levels and contributing to the development of both IFG and IGT. This finding aligns with a recent systematic review, saying high-starch diets increase insulin resistance and impair islet function, thereby elevating the risk of T2DM in women [54]. While adequate physical activity and dietary fiber intake generally improve insulin sensitivity [55], it may not fully overcome the metabolic burden and restore normal islet function imposed by a high-starch diet. This may explain why adequate physical activity fails to mitigate the metabolic effects of high-starch foods.

Surprisingly, in contrast to previous studies, we found that women with non-precarious employment were more likely to belong to the “Low Activity and Low Dietary Fiber Intake” group compared to those with precarious employment. In our study, non-precarious employees were primarily employed in indoor office or service jobs, which often left them with limited time and environmental opportunities for health-promoting activities. In contrast, women with precarious employment were mainly engaged in physically demanding roles, such as farming, which led to higher activity levels and contributed to a greater disparity between the two groups. Our study also found that women with inadequate external environmental resources had higher odds of engaging in the “Less Activity and Low Dietary Fiber Intake” group. As suggested by existing literature, limited access to recreational facilities, safe exercise spaces, and other community resources contributes to this pattern [56,57]. In the absence of structural support, individuals encountering numerous social disadvantages may experience difficulty in prioritizing lifestyle modifications, even when cognizant of their importance and benefits. Consistent with previous studies [58], women with a family history of diabetes were less likely to engage in the “Less Activity and Low Dietary Fiber Intake” group, possibly driven by greater awareness of their inherited metabolic risk and a heightened motivation to protective health behaviors [59].

Our research indicated that women with lower monthly family incomes or diminished psychological health are more likely to participate in the “Adequate Activity but High Starch Intake” category. This observation aligns with prior studies that reveal the strong influence of psychosocial determinants on personal food choices [60,61]. Firstly, for populations with limited financial resources, a significant factor affecting food choice is the cost of food [62]. Research has established that diets comprising lean meats, vegetables, and fruits generally tend to be associated with higher costs than energy-dense foods, such as desserts, salty snacks, candy, and sugary products [63]. Thus, low-income populations are more likely to choose inexpensive, energy-dense foods, which are often rich in sugars and fats, particularly refined carbohydrates. Secondly, psychological stress can limit cognitive resources, reducing the ability to plan long-term and engage in preventive health behaviors [64,65], particularly in underserved areas. Moreover, high-starch foods are typically calorie-dense and help activate brain reward circuitry, thereby counteracting the effects of negative emotional states by inhibiting elevated plasma corticosterone levels [66,67], which may explain the preference among women with poorer psychological health.

### 4.1. Implications

Our findings have several implications for clinical practice and research aimed at addressing high-risk behaviors in this vulnerable population to delay the progression from GDM to prediabetes status. Firstly, healthcare providers should conduct early screenings and education for women with prior GDM with a particular focus on the importance of physical activity levels (especially moderate- to vigorous-intensity) and carbohydrate intake (especially starch consumption). Screening and education on physical activity should focus on women who have non-precarious employment or no family history of diabetes. In contrast, dietary screening and education should prioritize women with low family income. Secondly, targeted interventions for women exhibiting high-risk behaviors, adding the components of psychological health and external environmental resources, are indicated. Specifically, creating an exercise-friendly environment may support behavior change. Improving the psychological health of the public population may enhance adherence to healthy eating habits.

### 4.2. Limitations

Firstly, although this study offers insights into the associations between behavior profiles and prediabetes status, its cross-sectional design precludes causal inference. In addition, the analysis was limited to the variables available at baseline, which may not fully capture all aspects of lifestyle-related factors. Secondly, some data (including physical activity and dietary intake) collected in this study were obtained through self-reported questionnaires, which could have introduced recall bias or response bias. Thirdly, within our three behavior profiles identified through LPA, one profile contained fewer participants than the others, which limited the comparability among profiles. Fourthly, the number of positive cases for IFG and IGT (used as dependent variables) in our binary logistic regression analysis was relatively low. Still, the β estimates from the post-hoc analysis remain robust and sufficient to support valid statistical inferences. Finally, as participants in this study were recruited exclusively from Hunan Province in south-central China, the generalizability of the findings to other regions may be limited.

## 5. Conclusions

Nearly 90% of the women exhibited high-risk behavior profiles, one of which is characterized by insufficient physical activity and dietary fiber intake, which is associated with increased odds of IFG. Conversely, another profile shows adequate physical activity and dietary fiber intake, combined with high starch intake, which is linked to greater odds of both IFG and IGT. Factors including occupation, family income, and diabetes history were associated with higher-risk profiles and may be considered when identifying subgroups within this vulnerable population. Strategies that enhance psychological health and improve external environmental resources could be considered in targeted lifestyle-related interventions.

## Figures and Tables

**Table 1:** Sample characteristics of women with prior gestational diabetes mellitus (N = 633).

Variables	n (%)	Mean (SD)
Trial		
Trial 1 ^a^	320 (50.55)	-
Trial 2 ^b^	313 (49.45)	-
Age (years)		
<35	422 (66.70)	32.78 (4.93)
≥35	211 (33.30)	
Months after delivery (months)	-	17.24 (13.58)
Marital Status		
Married	627 (99.05)	-
Never married/loss of spouse/history of divorce	6 (0.95)	-
Level of Education		
No finishing nine-year compulsory education	112 (17.69)	-
Finishing nine-year compulsory education	521 (82.31)	-
Occupation		
Non-precarious employment	312 (49.30)	-
Precarious employment	321 (50.70)	-
Monthly Family Income (USD)		
<416	103 (16.27)	-
≥416	530 (83.73)	-
Family History of Diabetes		
Yes	80 (12.64)	-
No	553 (87.36)	-
BMI ^c^		
<24.0	387 (60.70)	23.43 (3.66)
≥24.0	246 (39.30)	
Psychological Health	-	13.56 (2.02)
Social Relationship	-	14.86 (2.09)
Environmental Status	-	13.15 (1.92)
IFG ^d^		
Yes	67 (10.58)	-
No	566 (89.42)	-
IGT ^e^		
Yes	45 (7.11)	-
No	588 (92.89)	-
Precarious employment	321 (50.70)	-
Moderate to Vigorous Physical Activity (min/day)	-	56.64 (105.94)
Sitting (min/day)	-	247.08 (170.61)
Staple Foods (g/day)	-	335.79 (167.07)
Legumes (g/day)	-	60.32 (88.55)
Vegetables (g/day)	-	257.31 (319.54)
Fruits (g/day)	-	172.45 (197.87)

Note: ^a^ Trial 1, randomized controlled trials conducted between 2018 and 2019, ChiCTR1800015023. ^b^ Trial 2, randomized controlled trials conducted between 2022 and 2023, ChiCTR2200058150. ^c^ BMI, body mass index. ^d^ IFG, impaired fasting glucose. ^e^ IGT, impaired glucose tolerance.

**Table 2:** Indicators for each latent physical activity and dietary intake profile.

Models	AIC ^a^	BIC ^b^	aBIC ^c^	Entropy	LMR ^d^	BLRT ^e^	Proportion
Two-profile	27,741.610	27,839.521	27,769.673	0.918	0.030	0.000	0.926/0.074
Three-profile	27,637.144	27,770.658	27,675.411	0.856	0.104	0.000	0.804/0.133/0.063
Four-profile	27,537.988	27,707.106	27,586.460	0.744	0.305	0.000	0.188/0.627/0.019 /0.166
Five-profile	27,376.545	27,581.266	27,435.221	0.847	0.310	0.000	0.420/0.141/0.344 /0.016/0.079

Note: ^a^ AIC, Akaike Information Criterion. ^b^ BIC, Bayesian Information Criterion. ^c^ aBIC, adjusted-Bayesian Information Criterion. ^d^ LMR, Lo–Mendell–Rubin likelihood. ^e^ BLRT, bootstrapped likelihood ratio test.

**Table 3:** Descriptive statistics for the indicator variables that constituted the three profiles.

Variable	Less Activity and Low Dietary Fiber Intake Group (*n* = 509)	Adequate Activity but Low Dietary Fiber Intake Group (*n* = 84)	Adequate Activity but High Starch Intake Group (*n* = 40)	*p*
Staple foods (g/day)	330.61 ± 7.47	325.91 ± 16.38	433.36 ± 31.27	0.002
Legumes (g/day)	44.54 ± 2.42	67.21 ± 8.24	228.62 ± 26.93	0.000
Vegetables (g/day)	211.75 ± 10.44	264.96 ± 23.44	839.07 ± 118.52	0.000
Fruits (g/day)	150.41 ± 6.86	133.10 ± 14.89	549.48 ± 61.99	0.000
Dairy products (ml/day)	82.77 ± 5.08	92.82 ± 13.79	326.21 ± 78.11	0.000
Moderate to vigorous physical activity (min/day)	20.04 ± 1.37	267.39 ± 15.69	79.73 ± 17.09	0.000
Sedentary (min/day)	258.93 ± 8.10	193.37 ± 12.79	213.95 ± 22.72	0.003

**Table 4:** Binary logistic regression examining the association between three physical activity and dietary intake profiles and impaired fasting glucose and impaired glucose tolerance.

Variables	IFG ^a^				IGT ^b^			
	b	*p*	OR	95%CI	b	*p*	OR	95%CI
Reference group ^c^	-	-	-	-	-	-	-	-
Less Activity and Low Dietary Fiber Intake Group	1.333	0.029	3.792	(1.146, 12.543)	0.629	0.261	1.876	(0.626, 5.622)
Adequate Activity but High Starch Intake Group	1.844	0.012	6.321	(1.500, 26.639)	1.797	0.010	6.030	(1.530, 23.770)

Note: Adjusted for age, months after delivery, income, education, occupation, DM family history, BMI, waist circumference, and trial. ^a^ IFG, impaired fasting glucose. ^b^ IGT, impaired glucose tolerance. ^c^ Reference group is the “Adequate Activity but Low Dietary Fiber Intake Group”.

**Table 5:** Multinomial logistic regression for the related factors of the “Less Activity and Low Dietary Fiber Intake Group” and the “Adequate Activity but High Starch Intake Group”.

Variables	“Less Activity and Low Dietary Fiber Intake Group”				“Adequate Activity but High Starch Intake Group”			
	b	*p*	OR	95%CI	b	*p*	OR	95%CI
Occupation (Ref. Precarious employment)	-	-	-	-	-	-	-	-
Non-precarious employment	0.645	0.011	1.898	(1.171, 3.102)	0.517	0.201	1.676	(0.759, 3.701)
Monthly family income (Ref. ≥ 416 USD)	-	-	-	-	-	-	-	-
<416 USD	0.502	0.241	1.652	(0.714, 3.824)	1.339	0.027	3.817	(1.272, 11.450)
DM family history (Ref. With DM family history)	-	-	-	-	-	-	-	-
Without DM family history	0.816	0.028	1.991	(1.242, 4.121)	0.109	0.833	1.116	(0.404, 3.080)
Psychological aspect	0.032	0.652	1.033	(0.899, 1.187)	−0.337	0.008	0.714	(0.556, 0.918)
Environmental aspect	−0.254	0.004	0.776	(0.654, 0.920)	−0.046	0.735	0.955	(0.732, 1.246)

## Data Availability

The data are not publicly available due to ethical and privacy restrictions, as they contain sensitive personal health information. Anonymized data may be available from the corresponding author upon reasonable request.

## Supplementary Materials

The following supporting information can be downloaded at: https://www.mdpi.com/article/10.3390/nu18050812/s1, Table S1: Interaction analysis between lifestyle profiles and trial for IFG/IGT; Table S2: Binary logistic regression examining the association between three physical activity and dietary intake profiles and impaired fasting glucose and impaired glucose tolerance (Complete case, n = 616).

## Abbreviations

The following abbreviations are used in this manuscript:

GDM	Gestational diabetes mellitus
T2DM	Type 2 diabetes mellitus
IFG	Impaired fasting glucose
IGT	Impaired glucose tolerance
BMI	Body mass index
SEM	Socio-ecological model
IPAQ-SF	International Physical Activity Questionnaire Short Form
FFQ	Food Frequency Questionnaire
OGTTs	Oral glucose tolerance tests
FBG	Fasting plasma glucose level
2 h PG	2-hour plasma glucose level
