# Supplementary material for: Which Combined Profiles of Physical Activity and Dietary Intake Are Associated with Postpartum Prediabetes Status Among Women with Prior Gestational Diabetes Mellitus in Underserved Rural Areas of Central South China?
Source: Nutrients. 2026 Mar 1;18(5):812. doi: 10.3390/nu18050812 (PMC12986713; doi:10.3390/nu18050812)
Supplement: Supplementary file 1 [file nutrients-18-00812-s001.zip › nutrients-4129208-supplementary.pdf]

Table S1. Interaction analysis between lifestyle profiles and trial for IFG/IGT

	IFG[a]	IGT[b]
	p	*p*
Less Activity and Low Dietary Fiber Intake Group*trials	0.926	0.458
Adequate Activity but High Starch Intake Group*trials	0.481	0.873

Note: [a] IFG, impaired fasting glucose. [b] IGT, impaired glucose tolerance.

Table S2. Binary logistic regression examining the association between three physical activity and dietary intake profiles and impaired fasting glucose and impaired glucose tolerance. **(Complete case,n=616).**

**Variables**	**IFG[a]**				**IGT[b]**			
	b	*p*	OR	95%CI	b	*p*	OR	95%CI
Reference group[c]	-	-	-	-	-	-	-	-
Less Activity and Low Dietary Fiber Intake Group	1.287	0.035	3.621	(1.092, 12.002)	0.634	0.257	1.886	(0.629, 5.655)
Adequate Activity but High starch Intake Group	1.893	0.010	6.641	(1.567, 28.132)	1.871	0.008	6.496	(1.634, 25.815)

Note: Adjusted for age, months after delivery, income, education, occupation, DM family history, BMI, waist circumference, and trial. [a] IFG, impaired fasting glucose. [b] IGT, impaired glucose tolerance. [c] Reference group is the "Adequate Activity but Low Dietary Fiber Intake Group".
